# Extracellular Vesicle–associated GARP/TGFβ:LAP Mediates “Infectious” Allo-tolerance

**DOI:** 10.1097/TXD.0000000000001475

**Published:** 2023-05-24

**Authors:** William J. Burlingham, Ewa Jankowska-Gan, John H. Fechner, Christopher J. Little, Jianxin Wang, Seungpyo Hong, Miraf Molla, Jeremy A. Sullivan, David P. Foley

**Affiliations:** 1 Division of Transplantation, Department of Surgery, School of Medicine and Public Health, University of Wisconsin-Madison, Madison, WI.; 2 Wisconsin Center for NanoBioSystems, University of Wisconsin-Madison, Madison, WI.; 3 Pharmaceutical Sciences Division, School of Pharmacy, University of Wisconsin-Madison, Madison, WI.; 4 Department of Anesthesiology, School of Medicine and Public Health, University of Wisconsin-Madison, Madison, WI.

## Abstract

Here we test the hypothesis that, like CD81-associated “latent” IL35, the transforming growth factor (TGF)β:latency-associated peptide (LAP)/glycoprotein A repetitions predominant (GARP) complex was also tethered to small extracellular vesicles (sEVs), aka exosomes, produced by lymphocytes from allo-tolerized mice. Once these sEVs are taken up by conventional T cells, we also test whether TGFβ could be activated suppressing the local immune response.

**Methods.:**

C57BL/6 mice were tolerized by i.p. injection of CBA/J splenocytes followed by anti-CD40L/CD154 antibody treatment on days 0, 2, and 4. On day 35, spleen and lymph nodes were extracted and isolated lymphocytes were restimulated with sonicates of CBA splenocytes overnight. sEVs were extracted from culture supernatants by ultracentrifugation (100 000*g*) and assayed for (a) the presence of TGFβ:LAP associated with tetraspanins CD81,CD63, and CD9 by enzyme-linked immunosorbent assay; (b) GARP, critical to membrane association of TGFβ:LAP and to activation from its latent form, as well as various TGFβ receptors; and (c) TGFβ-dependent function in 1° and 2° immunosuppression of tetanus toxoid-immunized B6 splenocytes using trans-vivo delayed–type hypersensitivity assay.

**Results.:**

After tolerization, CBA-restimulated lymphocytes secreted GARP/TGFβ:LAP-coated extracellular vesicles. Like IL35 subunits, but unlike IL10, which was absent from ultracentrifuge pellets, GARP/TGFβ:LAP was mainly associated with CD81^+^ exosomes. sEV-bound GARP/TGFβ:LAP became active in both 1° and 2° immunosuppression, the latter requiring sEV uptake by “bystander” T cells and reexpression on the cell surface.

**Conclusions.:**

Like other immune-suppressive components of the Treg exosome, which are produced in a latent form, exosomal GARP/TGFβ:LAP produced by allo-specific regulatory T cells undergoes either immediate activation (1° suppression) or internalization by naive T cells, followed by surface reexpression and subsequent activation (2°), to become suppressive. Our results imply a membrane-associated form of TGFβ:LAP that, like exosomal IL35, can target “bystander” lymphocytes. This new finding implicates exosomal TGFβ:LAP along with Treg-derived GARP as part of the infectious tolerance network.

We previously reported that IL35 is not produced as an H_2_O-soluble cytokine like IL10 but rather is produced in a latent form associated with exosomes.^1^ The exosomes were produced by Tregs and Bregs in allo-tolerized B6 mice, and the IL35 subunits p35 and Ebi3 were not in a physiologic 1:1 ratio but rather in a latent, 2:1 ratio associated with the tetraspanin CD81. Because CD81 would coprecipitate with Abs to either subunit, and because CD81 association was abolished in the presence of β-mercaptoethanol, it suggested that the locus of binding was in the disulfide bonded region of CD81 known to be part of the large extracellular loop of the tetraspanin.^1,2^ Besides causing 1° IL35-based immunosuppression (IS), the Treg exosomes (a) induced a small subset of Foxp3^neg^ conventional T cells to become IL35-producers (iTr35)^3^ and (b) imparted a 2° suppressive phenotype to ~1% of naive T cells that uptake latent (2:1 subunit) IL35^+^exosomes and later display active (1:1 subunit) surface IL35.^1^ In mutant mice deleted for Ebi3 exclusively in Foxp3^+^ T cells, exosomes from tolerized animals failed to do the latter, indicating a key role for IL35 in the induction phase of 2° IS. Thus, IL35-subunit^+^ exosomes from Foxp3^+^ Tregs of tolerized mice appeared to play a key role in “infectious tolerance,” the Treg-based phenomenon originally described by Waldman and colleagues (“classic” paper, the senior (last) author of which is Herman Waldman).^4^ However, the possible participation of other exosomal-associated cytokines besides IL35 subunits in the effector phase of 2° IS was not assessed.^1^

Transforming growth factor (TGF)β1 has long been recognized as a key component of the Treg repertoire causing 1° IS. Nakamura et al^5^ found that TGFβ1 was present in a latent (latency-associated peptide [LAP]–associated) form on the surface of CD4^+^CD25^+^ Tregs but could suppress proliferation of CD4^+^CD25^neg^ T conventional cells (ie, be converted to active TGFβ1) in a cell–cell contact-dependent fashion. Although TGFβ1-associated exosomes have been reported in local tumor-specific IS in AML,^6^ no evidence has yet been produced associating the process of allo-specific IS via Treg-derived exosomes bearing latent TGFβ1.

Here we reassess the involvement of regulatory T-cell– and B-cell–derived TGFβ:LAP in allo-specific tolerance using a mouse model. We found an exosomal basis for natural TGFβ1-based immunoregulation due to secretion of TGFβ:LAP in association with small (30–200 A diameter) extracellular vesicles. Although the requirement for enzymatic processing of TGFβ:LAP to release active TGFβ for binding to its receptor has long been known, the basis of its activation on the surface of Treg cells, involving the interaction of surface TGFβ:LAP with glycoprotein A repetitions predominant (GARP) a leucine-rich repeat molecule of unknown function, has only recently come to light.^7,8^ We demonstrate the association of Treg/Breg-derived TGFβ:LAP and GARP with exosomes, a linkage that has been overlooked in prior analyses of induced allo-tolerance. A new model of allo-specific immunoregulation is proposed in which latent GARP/TGFβ:LAP joins several other known immunoregulatory products of suppressive T and B cells (IL35, CD39/73) that belong in the category of exosomal products and thus are ideally equipped to promote “infectious” tolerance.

## MATERIALS AND METHODS

### Mice

C57BL/6 mice (B6) [H2b], CBA [H2k], and DBA/2 [H2d] mice were purchased from Envigo (Indianapolis, IN). CB17 severe combined immunodeficient mice were obtained from UW-Madison Mouse Breeding Core. All mice were housed in a pathogen-free facility, and experiments were conducted in accordance with the NIH guidelines and after approval of the Institutional Animal Care and Use Committee.

### Tolerization

B6 mice 6 to 12 wk of age were tolerized by i.v. injection of 10 × 10^6^ x-irradiated CBA/J spleen cells on day 0, followed by i.p. injection of 125 μg of the anti-CD154 monoclonal antibody (MR1) on days 0, 2, and 4, as previously reported.^1,9^ On day 35, lymphocytes were isolated from extracted lymph nodes and spleen from tolerized mice and incubated with lysates of either (a) 10 × 10^6^ x-irradiated CBA/J splenocytes, (b) 10 × 10^6^ x-irradiated DBA/2 splenocytes, or (c) they were left unstimulated (media only). All cultures were done for 24 h in RPMI 1640 media containing penicillin/streptomycin and 10% exosome-free FCS.

### Isolation of Small Extracellular Vesicle Aka Exosomes

Culture supernatants were collected and subjected to serial centrifugation at 4 °C at increasing speeds (300*g*/10 min, 2000*g*/10 min, and 10 000*g*/30 min) to remove cells, dead cells, and cell debris. After that, supernatants were filtered (0.45 µm) and subjected to 100 000*g* ultracentrifugation for 2 h at 4 °C. Supernatants were collected as an extracellular vesicle–free fraction, the pellets were resuspended in 4 mL of PBS, and another 100 000*g* ultracentrifugation for 2 h at 4 °C was performed. The pelleted exosomes were resuspended in PBS and analyzed by Nanosite Tracking Analysis (Malvern Instruments, Malvern, United Kingdom), enzyme-linked immunosorbent assay (ELISA), and transmission electron-microscopy.

### ELISA

The ELISA for exosome-associated TGFβ1 was done using a modification of the methods of Logozzi et al^10^ and Sullivan et al.^1^ Half-area, high binding microtiter plates were coated with capture antibodies to LAP-TGF-β1 (BioLegend, Clone TW7-16B4); LTBP I, II, or III (Bioss Antibodies); GARP (Biolegend F011-5); or TGFβ1 (Abcam) at 10 µg/mL each in 10 mmol/L TRIS pH9 and incubated overnight at 4 °C. After blocking plate with 2% BSA in PBS for 3 h at room temperature, tested samples were added, and plates were incubated overnight at 4 °C. Wells were washed, in PBS/0.1%BSA, and bound target antigen was detected with the following biotinylated anti-tetraspanin monoclonal antibodies: anti-CD81 (Thermo Fisher, MA5-1793), anti-CD9 (Biolegend, MZ3), or anti-CD63 (Biolegend, NVG-2) at 1 µg/mL concentration. After 3 h of incubation at room temperature, wells were washed, and HRP-Streptavidin (Biolegend) was added at 1:1000 dilution. After 45 min incubation, wells were washed, and a solution containing 3,3′,5,5′-tetramethylbenzidine (TMB) was added. The reaction was stopped after 10 min with TMB stop solution, and color development was read at 450 nm.

### Functional Assay

To assess 1° IS, tetanus toxoid (TT)–immunized SC and LNC from B6 mice were exposed to small extracellular vesicles (sEVs) that had been purified from culture supernatants as described above. After a brief coincubation, mixtures of sEV and TT-sensitized cells (5 × 10^6^) were injected into the footpads of naive B6 mice for the trans-vivo delayed type hypersensitivity (tvDTH) assay.^11^ Controls were media alone, TT alone, and TT plus sEV from DBA/J antigen-restimulated cultures. To assess the cytokine basis of sEV-based 1° IS of the TT response, footpad swelling in the presence of sEVs from the CBA/J-restimulated culture was evaluated in the presence of normal goat IgG, anti-IL10, anti-IL35 [anti-p35 + anti-Ebi3], and anti-TGFβ.^1^

To assess 2° IS, normal T cells were purified from naive B6 lymphocytes. The cells were then incubated overnight with sEVs from CBA/J-restimulated cultures of cells from CBA/J-tolerized mice. As controls, we also treated B6 naive T cells with sEVs isolated from DBA/2 (third party)-restimulated cultures. After being washed 2 times to remove any nonadsorbed exosomes, exosome-treated B6 T cells were added to TT-sensitized B6 SC at a 1:1 ratio, mixed with TT, and injected into the mouse footpad along with normal mouse IgG, anti-IL35, or anti-TGFβ. The 2° suppression of TT response in tvDTH was then evaluated under each condition.^1^

## RESULTS

**FIGURE 1. Experimental design and tetraspanin content of TGFβ1-containing EV.**
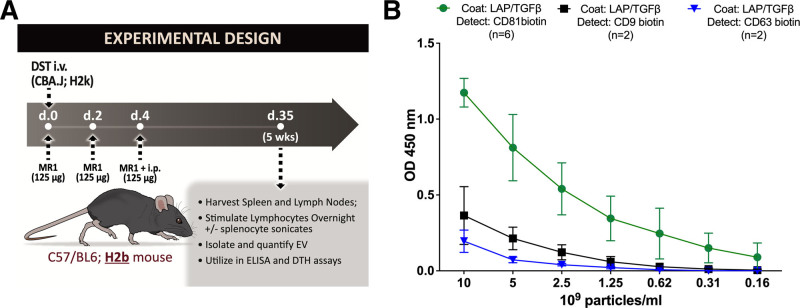
A, Diagram of exosome generation. B, Detection of LAP-TGFβ1 on CD81^+^, CD9^+^, and CD63^+^ exosomes produced by CBA/J-tolerized B6 lymphocytes in vitro in response to tolerizing alloantigen. DTH, delayed type hypersensitivity; ELISA, enzyme-linked immunosorbent assay; EV, extracellular vesicle; LAP, latency-associated peptide; TGF, transforming growth factor.

Spleen and lymph nodes containing T and B lymphocytes of tolerized B6 mice were extracted on day 35 and restimulated with sonicates of CBA splenocytes for 24 h. Soluble donor antigen, the focus of much of our laboratory work investigating donor specific regulation using the trans-vivo DTH, can be processed by APCs within the tolerized splenocytes for subsequent presentation to antidonor effector and regulatory T cells also within the splenocyte population. After culture, the cells and media are collected and then undergo sequential centrifugation to remove cells and large debris and isolate sEVs/exosomes (Figure 1A).

We then assayed for tetraspanin-associated TGFβ:LAP by ELISA on exosomes, as well as exosome-depleted ultracentrifuge supernatants produced in vitro. Ultracentrifuge supernatant was completely negative for tetraspanins (data not shown). In the ultracentrifuge pellets, we found an abundant signal for TGFβ:LAP on CD81^+^ exosomes and a much lower TGFβ:LAP signal on CD9^+^ and CD63^+^ exosomes (Figure 1B). This agrees with our previous finding of CD81 as the predominant tetraspanin used in the production of exosomes carrying IL35 subunits from Tregs and Bregs in the same allo-tolerance model. The results shown represent the mean ± SE of 3 separate tolerizations, indicating a consistent bias toward CD81^+^ exosomes in the TGFβ-LAP response.

We next attempted to detect GARP, the key to membrane association of TGFβ:LAP, on the same exosomes. Because GARP is required for membrane association and activation of TGFβ by αVβ8 integrins, our purpose here was to determine if there was GARP available on the same exosomes to enable the exosome-acquiring B or T lymphocytes to remove the LAP-coating and expose active TGFβ1 that could bind the TGFβ1 receptor and initiate intracellular signals.

**FIGURE 2.**
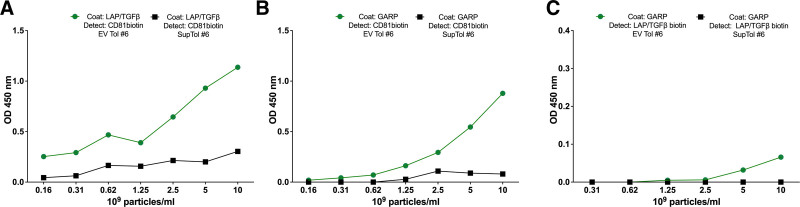
ELISA showing presence of GARP on the exosomes released by d.35 Tregs on rechallenge. In each panel, the green line indicates the exosome fraction, whereas the black line is the exosome-free supernatant fraction. Antibodies to (A) anti-LAP/TGFβ1 coating with anti-CD81 detection and (B) anti-GARP coating with anti-CD81 detection and anti-GARP coating with anti-LAP/TGFβ1 detection. ELISA, enzyme-linked immunosorbent assay; EV, extracellular vesicle; GARP, glycoprotein A repetitions predominant; LAP, latency-associated peptide; TGF, transforming growth factor.

As shown in Figure 2, the same CD81^+^ exosome preparation that is TGFβ:LAP^+^ (Figure 2A) also contained GARP^+^ extracellular vesicles. We could detect GARP both by capture with anti-GARP antibodies followed by detection with CD81 (Figure 2B) and by capture with anti-TGFβ:LAP and detection with anti-GARP (Figure 2C). However, the latter detection signal was much lower, suggesting that the antibodies to GARP and to TGFβ:LAP had interfered with each other, as expected given the proximity of GARP to TGFβ:LAP in the latent GARP/TGFβ:LAP complex.^12^

Besides establishing the presence of GARP, required for the process of TGFβ1 activation, we assessed TGFβ1 receptors present on exosomes (**Figure S1, SDC**, http://links.lww.com/TXD/A521). Indeed we found low levels of TGFβ1 receptors I, II, and III by ELISA on exosomes produced after restimulation of tolerized splenocytes.

**FIGURE 3.**
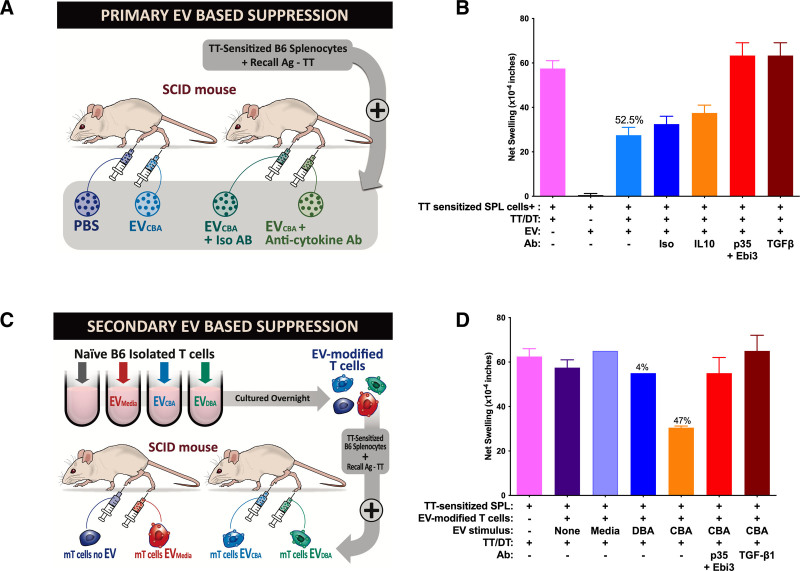
Inhibition of trans-vivo DTH response to TT by day 35 exosomes. A, Diagram of 1° IS assay. B, Results of 1° S by direct addition of exosomes and effects of added antibodies. Results shown are the mean (±SE) of 2 separate experiments. C, Diagram of 2° IS assay. D, Results of 2° IS assay after modification of naive B6 T lymphocytes by exosomes followed by assay of modified T cells as inhibitors of a footpad anti-TT DTH. Controls included addition to normal B6 T cells of exosome-free RPMI, plus exosomes from media-, and DBA-stimulated coculture. DTH, delayed type hypersensitivity; EV, extracellular vesicle; IS, immunosuppression; SCID, severe combined immunodeficient; TT, tetanus toxoid.

Although present on exosomes released by allo-specific regulatory cells only in an inactive, “latent” form, both IL35 and TGFβ1 became active in 1° IS (Figure 3A). We measured 1° suppression by first showing a strong anti-TT tvDTH response of splenocytes from tetanus-sensitized B6 mice transferred into the footpads of CB17-severe combined immunodeficient mice (Figure 3B, pink bar). We then observed a >50% reduction in swelling response (Figure 3B, light blue bar) when “day 35 sEVs” were added to B6 effectors and TT before footpad injection. Complete reversal of this suppression by either IL35- or TGFβ1-specific antibodies was seen, indicating the critical role of these cytokines in exosome-mediated immune suppression. On the other hand, IL10, previously shown to be a component of 1° IS using whole splenocytes but not a factor associated with the exosome fraction of Treg culture supernatant,^1^ was not involved in exosome-mediated IS, as the addition of anti-IL10 had little or no effect, similar to the addition of normal mouse IgG (Figure 3B, compare dark blue and orange bars).

We next evaluated the role of the 2 “latent” exosomal cytokines (IL35, TGFβ1) in the phenomenon of 2° IS (Figure 3C). Rather than directly exposing TT-specific T cells to exosomes as was done in Figure 3A and B, instead, an overnight incubation of normal B6 T cells with sEVs/exosomes was done. This step decorates naive T cells with exosomes from the day 35 cultures.^1,13^ Each sample of sEV-treated T cells was then washed 2 times and added to TT-sensitized B6 splenocytes before footpad injection. We could then measure 2° inhibition of a tvDTH (footpad swelling) response, using antibodies to block any exosome-acquired cytokines. Clearly shown in Figure 3D is the antigen specificity of exosome induction; CBA-lysate-induced exosomes were able to transfer 2° suppression (47% inhibition; orange bar), whereas DBA (third party; blue bar) lysate-induced exosomes did not (4% inhibition). As to the mechanism of 2° regulation, anti-IL35 (anti-Ebi3 plus anti-p35) antibodies were found to completely reverse the 2° IS (Figure 3D, red bar), as expected based on our previous report.^1^ What is new here is that anti-TGFβ1 also restored maximal footpad swelling. This suggests that both exosomally associated latent cytokines (inactive IL35 and LAP-TGFβ1: GARP) were capable of being acquired, undergoing activation, and mediating 2° IS. We should note that because 2° IS involves treatment of normal B6 (ie, “bystander”) T cells with sEVs from CBA-tolerized (day 35 restimulated) B6 lymphocytes, it is a genuine test of “infectious” tolerance, the recruitment of naive T cells into the suppression process.^13^

**FIGURE 4.**
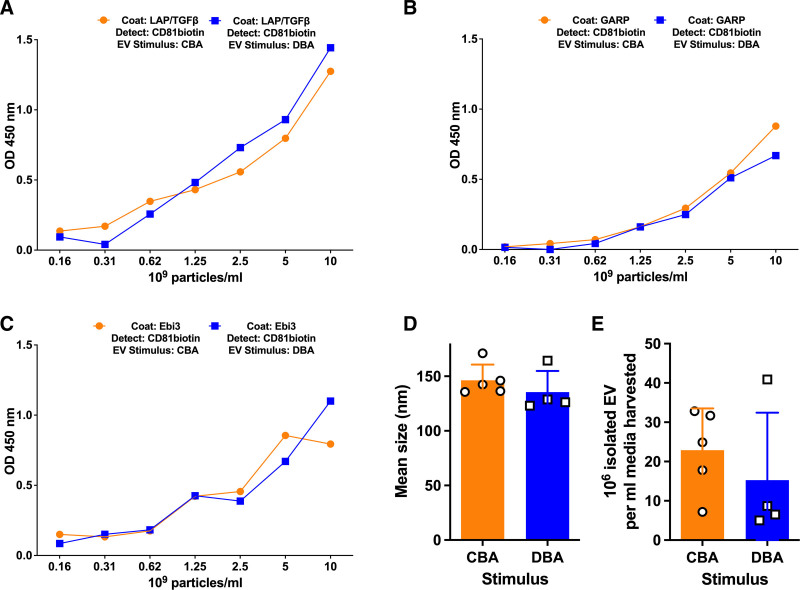
Comparison of exosomes in CBA/J- and DBA/2-antigen-restimulated cultures. As shown here, despite a clear difference in functional suppression (Figure 3B), we could detect no significant differences between CD81^+^ CBA/J- and DBA/2-restimulated d.35 exosomes based on (A) LAP-TGFβ1, (B) GARP, or (C) Ebi3 content, nor were their (D) mean size or (E) recovered concentration different. GARP, glycoprotein A repetitions predominant; LAP, latency-associated peptide; TGF, transforming growth factor.

Although the induction of exosomes causing 2° IS was quite specific to the tolerizing alloantigen (note: CBA/J, but not DBA/2, lysates induced sEVs with 2° IS properties; Figure 3C and D), we were surprised to find that this allo-specificity did not hold at the ELISA detection level. As shown in Figure 4, neither the number of particles/milliliters nor the relative size of the exosome particles containing CD81, TGFβ:LAP, or the Ebi3 component of IL35 appeared to differ between CBA/J- and DBA/2-antigen-restimulated cultures. Thus, some other factor, not detected by the ELISAs, must account for the clear allo-specificity (Figure 3D) of the 2° IS by the Treg and Breg exosomes.

## DISCUSSION

The results presented here clearly establish that TGFβ in its latent LAP-associated form is secreted by regulatory lymphocytes in allo-tolerized mice in association with exosomes. Two paradoxes need to be explained: (1) that despite the fact that TGFβ in exosomes was present only in a latent form, the active form became available in both 1° and 2° IS; and (2) that in transgenic Ebi3 knockout mice lacking IL35 expression in Foxp3^+^ cells, no 2° IS was observed,^1^ yet in wild type mice, both IL35 and TGFβ were apparently components of the 2° suppressive mechanism of exosomes.

**FIGURE 5.**
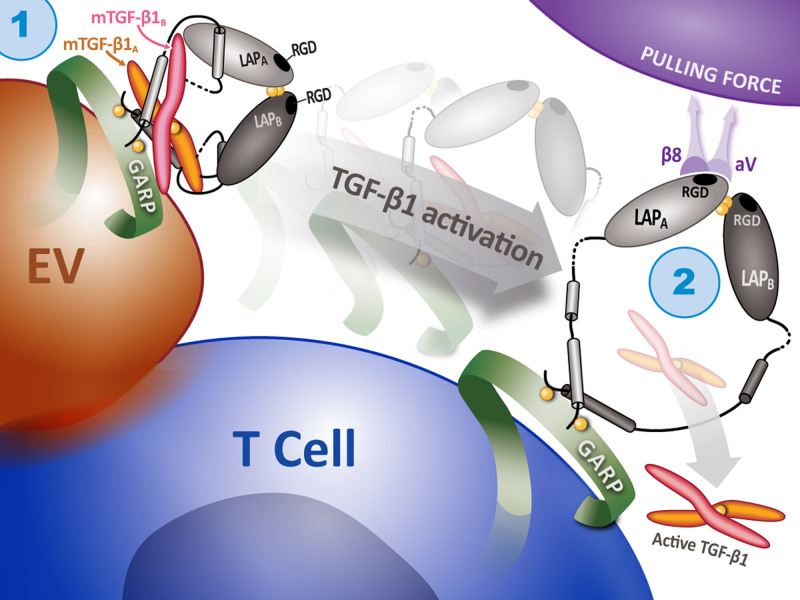
A proposed model of acquisition and activation of the GARP:LAP:TGFβ1 complex from a Treg-derived exosome based on Lienart et al.^12^ Arrows indicate how a pulling force provided by another cell activates the TGFβ1 from the exosomal precursor. EV, extracellular vesicle; GARP, glycoprotein A repetitions predominant; LAP, latency-associated peptide; TGF, transforming growth factor; RGD, Arginine:Leucine:Aspartic Acid motif.

As to the first question, the mechanism of binding of TGFβ:LAP to the surface of the Treg cell had been a conundrum puzzling investigators for years after its description by Strober and colleagues^5^ until the breakthrough discovery of the association of TGFβ:LAP with the GARP molecule, a membrane-bound protein.^14–16^ Our data (Figure 2) indicate that Treg- and Breg-derived exosomes also contain GARP. The work of Sophie Lucas and colleagues (paper from the laboratory of Sophie Lucas)^12^ has elucidated the structure of the GARP/TGFβ1:LAP complex in both the compressed/latent and extended/activated form. Figure 5 shows a possible model that reconciles our exosomal data (Figure 2B) with this proposed activation mechanism. As shown here, TGFβ1 activation is initiated once the GARP/TGFβ1:LAP is bound to the exosome surface and becomes incorporated into the surface membrane of the T cell. An opposing cell bearing the αVβ8 integrin binds to the arginine:leucine:aspartic acid (RGD) motif of the LAP molecule (black zone on LAP_A_ and LAP_B_ subunits). A pulling force is then applied from the opposing cell; this is complemented, by either an equivalent pulling force or simply resistance, from the exosome-acquiring T cell. The result is thought to be either the release of free TGFβ1, as proposed by Lienart et al^12^ and depicted in Figure 5, or the exposure of TGFβ1 to its receptor while still loosely bound to the GARP/LAP complex.^17^

The discovery of the role of the GARP/TGFβ1:LAP interface, allowing access for the αVβ8 integrin on an opposing cell surface,^7,8^ as well as the proposed role of the large TGFβ1-binding protein (LTBP1)-TGFβ1:LAP interface, allowing access to matrix-sequestered latent TGFβ1 via αVβ6 integrin,^18^ suggests that activation of TGFβ1 can only occur by the application of force between adjacent cells or between cells and the surrounding matrix. These discoveries have allowed for novel approaches to interfering with TGFβ1-mediated immune regulation by Treg cells without interfering with widespread TGFβ-mediated effects necessary for normal tissue stability. So, for example, by pinpointing the exact GARP/TGFβ:LAP interface with mAb therapy, it is now possible to interfere with the local immune-suppressive TGFβ1-mediated effects of Tregs within a tumor, thus promoting PD-1/PD-L1–based immune activation in cancer immunotherapy.^8^ This also means that during transplantation, the gathering of Tregs and Bregs within the graft and their exosomal TGFβ:LAP/GARP release may benefit the patient undergoing withdrawal or minimization of immunosuppressive drug therapy by propagating local IS.

As to the second issue, because of our previous report,^1^ we were surprised to find that both IL35 and TGFβ1 are involved in 2° IS, the curious ability of naive T cells to suppress immune function after exposure to Treg-derived exosomes. It now appears that in wt mice, IL35 is only one of several immune-suppressive cytokines/enzymes (eg, CD39/CD73) present on such exosomes.^19^ Therefore, although Treg-derived IL35 was necessary for induction of 2° IS by exosomes in tolerized mice,^1^ other components of the exosomes from a tolerized mouse, besides IL35, may participate in the effector phase of 2° IS. One such cytokine transferred to naive B6 T cells by exosome incubation is clearly the latent form of TGFβ1, which becomes activated to participate in 2° IS (Figure 3C and D). One possible explanation for the discrepancy between the induction and effector phase of immune suppression is that IL35 is necessary to increase αVβ8 integrin expression, as the latter integrin is known to be needed for TGFβ1 activation at the cell surface.^20^

In transplantation, the advantage provided by exosomal TGFβ1:LAP is the potential of continuous local transfer of inactive TGFβ1 from graft-infiltrating regulatory cells to other lymphocytes entering the graft. If accompanied by the cotransfer of GARP, as our data (Figure 2) indicate, it would provide the means for TGFβ1 activation at the surface of the exosome-acquiring cell and therefore another component of 2° IS by Treg (and Breg) exosomes. The data provided here illuminate an additional aspect of the infectious tolerance^4^ mechanism in transplantation.

## Supplementary Material


