# Supplementary material for: Extracellular Vesicle–associated GARP/TGFβ:LAP Mediates “Infectious” Allo-tolerance
Source: Transplant Direct. 2023 May 24;9(6):e1475. doi: 10.1097/TXD.0000000000001475 (PMC10212611; doi:10.1097/TXD.0000000000001475)
Supplement: Supplementary file 1 [file txd-9-e1475-s001.pdf]

Supplement

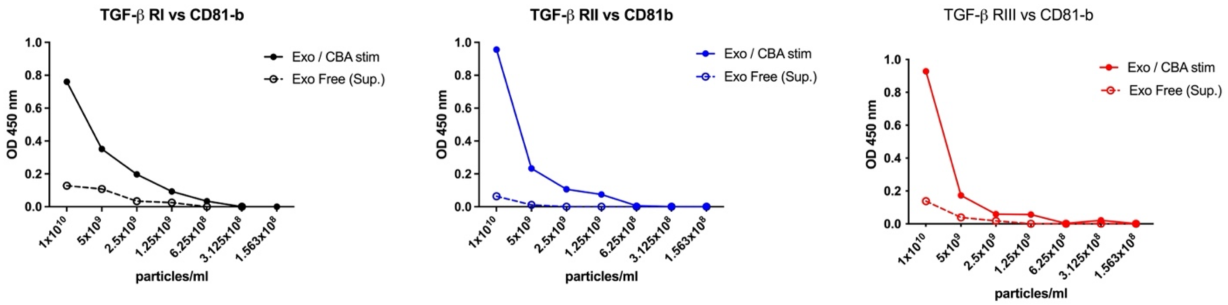

Supplemental Figure S1 shows that the same CD81+ exosomes bearing latent TGFβ also contained low levels of the 3 known receptors for active TGFβ. The signal for TGFβ-RI was slightly higher than that of TGFβ-RII and RIII.
